# Supplementary material for: Identification and Characterization of Common Bean (Phaseolus vulgaris) Non-Nodulating Mutants Altered in Rhizobial Infection
Source: Plants (Basel). 2023 Mar 14;12(6):1310. doi: 10.3390/plants12061310 (PMC10059843; doi:10.3390/plants12061310)
Supplement: Supplementary file 1 [file plants-12-01310-s001.zip › plants-2235049-Table S1.pdf]

Table S1. Segregation ratio of wt nodulation (wt) vs. mutant non-nodulation (*nnod*) (wt : *nnod*) phenotype in M5 plants derived from selected mutant lines

**Mutant line ID**	**Number of M5 plants analyzed derived from M4**	**wt : *nnod***	**Number of M5 plants analyzed derived from M4**	**wt : *nnod***
08IS-0647	12	6 : 1	10	6 : 1
08IS-0656	12	6 : 1	10	6 : 1
08IS-0771	12	2 : 1	10	2 : 1
08IS-0960	11	5 : 1	10	2 : 1
08IS-1001	9	7 : 1	10	5 : 1
08IS-1020	12	6 : 1	12	6 : 1
08IS-1022	12	4 : 1	10	2 : 1
08IS-1406	9	5 : 1	9	4 : 1
**08IS-1895**	**12**	**2 : 1**	**11**	**3 : 1**
08IS-1927	12	6 : 1	12	wt
**08IS-2114**	**12**	**4 : 1**	**11**	**2 : 1**
**08IS-2353**	**11**	**4 : 1**	**11**	**3 : 1**
08IS-2401	12	4 : 1	12	wt

The mutants that were characterized for this work are written in bold. The mutant lines: 08IS-0508, 08IS-0557, 08IS-0582, 08IS-0586, 08IS-0594, 08IS-0602, 08IS-0604, 08IS-0607, 08IS-0615, 08IS-0624, 08IS-0625, 08IS-0661, 08IS-0664, 08IS-0665, 08IS-1021, 08IS-1038, 08IS-1828, 08IS-1959, 08IS-2111, 08IS-2212, 08IS-2360, 08IS-2375 and 08IS-2398 were also screened but these showed a wt phenotype in every plant.
