# Supplementary figures and images for: Identification and Characterization of Common Bean (Phaseolus vulgaris) Non-Nodulating Mutants Altered in Rhizobial Infection
Source: Plants (Basel). 2023 Mar 14;12(6):1310. doi: 10.3390/plants12061310 (PMC10059843; doi:10.3390/plants12061310)

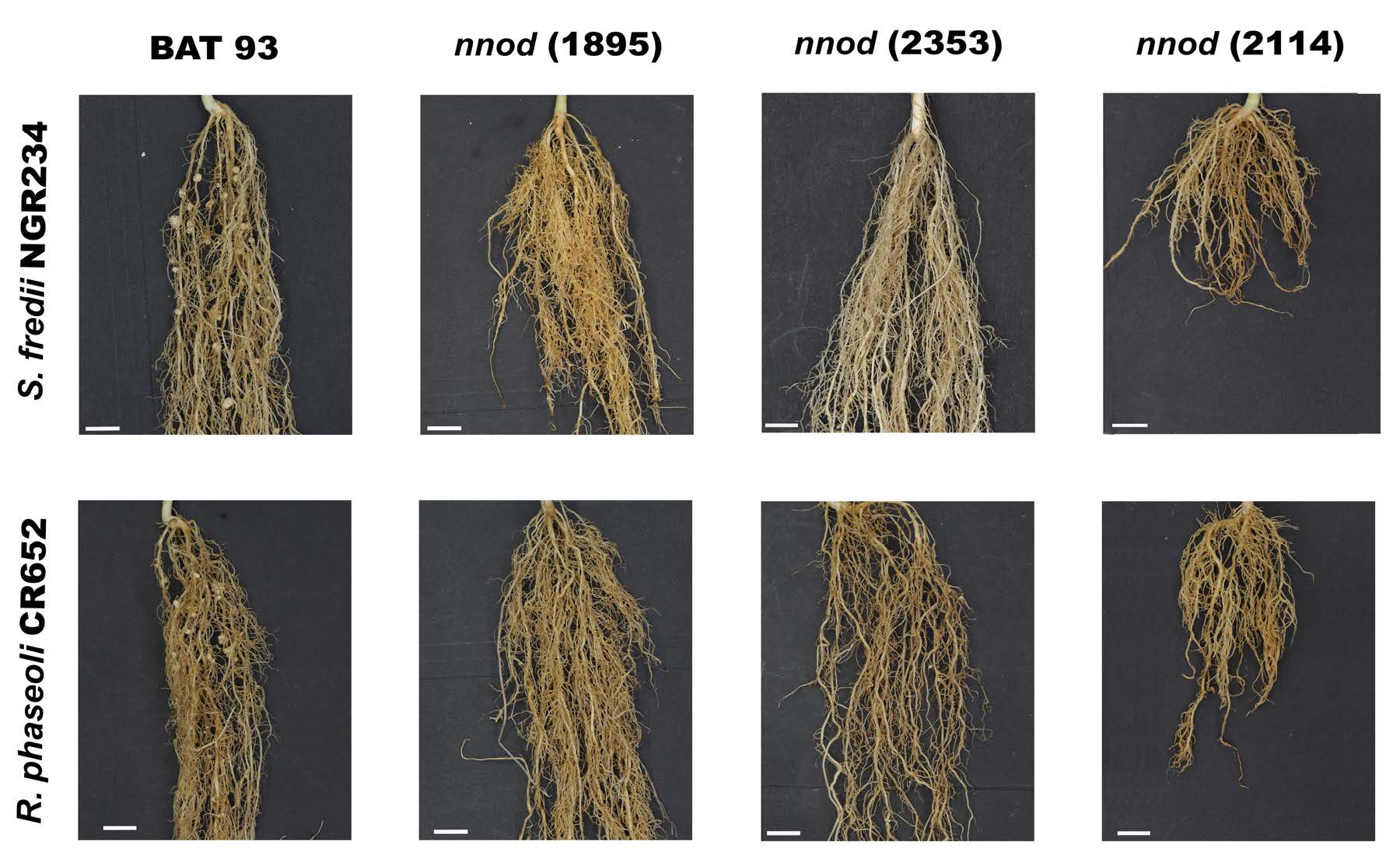

Supplement: Supplementary file 1 [file plants-12-01310-s001.zip › Supplemental Figure S1.jpg]
